# Reliability and validity of a widely-available AI tool for assessment of stress based on speech

**DOI:** 10.1038/s41598-023-47153-1

**Published:** 2023-11-18

**Authors:** Batul A. Yawer, Julie Liss, Visar Berisha

**Affiliations:** Arizona State University, Tempe, USA

**Keywords:** Health care, Neurology

## Abstract

Cigna’s online stress management toolkit includes an AI-based tool that purports to evaluate a person’s psychological stress level based on analysis of their speech, the Cigna StressWaves Test (CSWT). In this study, we evaluate the claim that the CSWT is a “clinical grade” tool via an independent validation. The results suggest that the CSWT is not repeatable and has poor convergent validity; the public availability of the CSWT despite insufficient validation data highlights concerns regarding premature deployment of digital health tools for stress and anxiety management.

## Introduction

Psychological stress has been linked to numerous health problems worldwide, including cardiovascular disease, hypertension, and depression^1,2^. Traditionally, psychological stress has been monitored via patient-reported questionnaires, like the Perceived Stress Scale (PSS). The PSS is a well-established questionnaire for measuring stress, with high reliability and validity^3–6^. It has been widely used as a reference for studying other modalities of stress measurement (e.g., cortisol concentration^7–9^) and for measuring the effectiveness of stress management techniques^10^. More recently, there has been growing interest in AI-based digital health tools for assessment of stress, depression, and anxiety^11,12^. The Cigna StressWaves Test (CSWT) is a publicly available proprietary AI tool used for analysis of psychological stress based on the acoustic features of speech and semantic features of the words spoken from a user speech sample^13,14^. To our knowledge, no published validation data exists for it despite its wide availability and integration into a broader offering for managing stress and anxiety by a global health services company. This paper presents independent validation data for the CSWT.

Speech-based artificial intelligence (AI) models have been proposed to monitor a speaker’s stress level, but their validation remains limited compared to standard instruments. While there is a body of scientific literature around speech production under stress^15^, little has been done in terms of model validation^12^. In contrast, other scales like the PSS demonstrate high internal consistency, temporal stability, and construct validity, as evidenced by high intra-class correlations and correlations with other psychometric scales^3–6^.

Despite the lack of independent validation, the CSWT asserts "clinical-grade” performance^16^, utility as a “stress diagnostic tool”, and design for “regular check-ins to retake the test”^17^; all this connotes high reliability and validity^18^. In this paper, we assess these claims by examining the CSWT’s test–retest reliability and validity relative to the PSS.

## Results

Sixty participants (36 F, 24 M) completed the CSWT twice during the same session (for reliability analysis) and the PSS once (for validity analysis). The PSS and CSWT were counterbalanced. Table 1 shows descriptive statistics for the stress scales and the participants’ age.

**Table 1:** Descriptive statistics of the study sample (N = 60, 36 F, 24 M).

Variable	M (SD)	Range—ordinal (min:max)	Median—ordinal	Mode—ordinal
Age	26.35 (8.57)	–	–	–
Perceived Stress Scale (PSS)	17.12 (5.23)	2 (1:3)	2	2
Cigna SWT (1)	13.50 (5.96)	2 (1:3)	1	1
Cigna SWT (2)	12.78 (5.79)	2 (1:3)	1	1

The PSS and the Cigna SWT provide both continuous and ordinal outputs. The mean and standard deviation correspond to the continuous output whereas the range, median, and mode correspond to the ordinal outputs.

### Repeatability results

**Figure 1:**
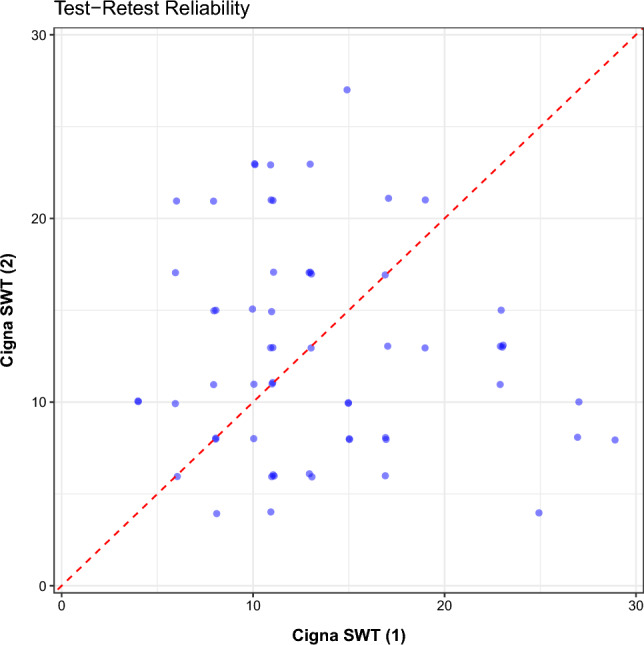
The test–retest plot for the Cigna StressWaves test. Each pair of samples was measured during the same session. The intra-class correlation of the test is ICC = −0.106, p > 0.05.

The test–retest reliability, as measured by the intra-class correlation between the two full-scale outputs of the two CSWT administrations, indicated that the test was not repeatable, (ICC = −0.106, p > 0.05). This is shown in Fig. 1, which displays the full-scale outputs from the two CSWT administrations and the line *x* = *y*. The reliability results did not change when the ordinal outputs were compared. Results of Cohen’s Kappa between the CSWT ordinal ratings showed no significant relationship between the two administrations of the test (κ = −0.176, p > 0.05).

### Validity results

**Figure 2:**
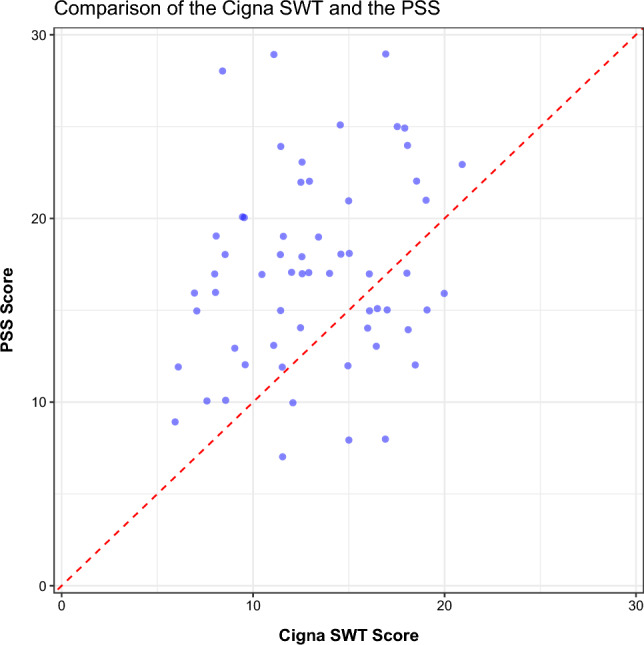
Convergent validity plot for the Cigna StressWaves test relative to the Perceived Stress Scale. The correlation between the two scores is r = 0.200, p > 0.05.

Convergent validity was assessed by examining the correlation between the CSWT and the PSS full-scale scores and Cohen’s Kappa between the ordinal ratings. Results showed that the CSWT score (average of two test administrations) was not significantly correlated with the PSS (r = 0.200, p > 0.05). This is shown in Fig. 2, which displays the full-scale averaged outputs from the CSWT and the PSS. The validity results did not change when the ordinal outputs were compared. Results of the Cohen’s Kappa between the PSS ordinal ratings and the first and second CSWT administrations’ ordinal ratings showed no relationship (PSS vs. CSWT (1): κ = 0.127, p > 0.05; PSS vs. CSWT (2): κ = 0.12, p > 0.05).

We further assessed convergent validity by using both CSWT administrations to predict the PSS via multiple linear regression. Results indicated that there was a collective significant effect between the two administrations of the CSWT and the PSS, (F(2, 57) = 3.184, p < 0.05, Adjusted R^2^ = 0.069). That is, when using both CSWT administrations to predict the PSS, the model explains 6.9% of the variance in the PSS. In totality, these results suggest poor convergent validity of the CSWT relative to the PSS.

## Discussion

The CSWT is presented as a clinical grade tool and offered as a part of a broader stress management toolkit. The results herein fail to support the claim of clinical grade performance and raise questions as to whether the tool is effective at all. This external validation study found that the CSWT has poor test–retest reliability and poor validity. The convergent validity results suggest that the CSWT has limited agreement with the PSS. Even when both test administration results were used to predict the PSS using linear regression, the model explained only 6.9% of the variance in the PSS. Our findings align with previously-highlighted concerns that widespread adoption of AI technologies are being prioritized over ensuring the devices work^12^. The widespread availability of this tool for stress and anxiety management, particularly through a large insurance company, may lead users to rely on it for assessing psychological stress levels and making healthcare decisions. As a result, misleading or inaccurate results can contribute to a variety of negative consequences, such as inappropriate treatment, wasted resources, increased anxiety, or false reassurance.

Additionally, the CSWT’s interpretations of a respondent’s results are not limited to state psychological stress (acute, transient) that the respondent may be feeling at the time they complete the test; rather, their interpretations extend to trait psychological stress (e.g., "you’re under a balanced level of pressure day-to-day"). Extrapolating trait psychological stress from a single 1-minute speech sample is unlikely to be feasible, even if the CSWT scores were valid and reliable in assessing state psychological stress.

The results of this study serve as an example of the fallacy of AI functionality^19^, where companies deploy AI tools under the assumption that they work but without requisite validation data. In healthcare, the mechanisms for verifying claims about a device’s functionality are well-established^20,21^. Online digital health tools should not be exempt from this level of scrutiny. Any deployed digital health tools should be grounded in verifiable claims with published evidence of functionality. In the absence of such data, these tools should not be made widely available.

The results of this study further highlight the previously documented challenges associated with building speech-based measures of health^22^. The within-subject and between-subject variability associated with speech production makes robust cross-sectional prediction challenging. The lack of transparency with the CSWT (in terms of validation data, functionality, and contact information) also makes it difficult to evaluate model quality. While the CSWT does not make public the information regarding the underlying model (i.e., what acoustic and semantic features are used), the most common approach to building clinical speech models is supervised learning^23^. This is where the authors train high-dimensional models to predict a clinical variable of interest. It’s been documented that models trained under this paradigm are less likely to generalize^22,23^, which can be partially attributed to the variability of commonly used features in the clinical speech literature^24^. We posit that feature variability imposes inherent limits on *any* algorithm’s ability to accurately predict complex health constructs (i.e. psychological stress, depression, anxiety) directly from speech. It is important to note that this limitation cannot be overcome by collecting larger training data or using more complex models as it is a property of the variability associated with human speech production.

## Method

### Participants

Our study included 60 participants over the age of 18, recruited at Arizona State University. The research was approved by the institutional review board of Arizona State University (IRB #00016588). The methods were carried out in accordance with the approved IRB and informed consent was collected from all participants via an online form prior to the start of the experiment. The inclusion criteria for the study were broad: all participants who spoke English and were over the age of 18. The Cigna StressWaves website indicates that the device can be used by all English speakers, even if English is not their primary language^13^.

### Test setting

All participants used the same equipment (i.e., Logitech H390 Wired Headset connected to a Dell computer) and conducted the experiment in a quiet laboratory environment. Participants were not shown their CSWT stress scores.

### The Cigna StressWaves test

The CSWT is presented as a clinical-grade tool for assessing a patient’s psychological stress level based on analysis of their speech. The user is prompted to select a question and provide a response lasting at least 60 seconds. In this study, we asked participants to perform the test twice to evaluate test–retest reliability. Each participant responded to one of the eight prompts on two consecutive administrations of the test during the same session (all sessions lasted 10 min or less). The participant was able to freely choose any of the eight prompts for each of the two sessions. Only one participant chose the same prompt twice. The tool provides an ordinal scale output (i.e., low, moderate, or high) and a full-scale score presented on a gradient scale. Each participant also completed the 10-question PSS. The PSS is also scored numerically on a full scale and on a three-level ordinal scale (i.e., numerical range from 0 to 40; low, moderate, and high)^25^. The order of PSS and CSWT was randomized across participants.

### Statistical analysis

The primary analysis in the study is the test–retest reliability, measured via the intra-class correlation (ICC) between the first and second administration of the CSWT. The secondary analysis is the evaluation of validity of the CSWT relative to the PSS, measured via the correlation between the PSS score and the average of the two CSWT scores. We average the scores between the two administrations to reduce CSWT variability. We use the PSS as a comparison as it produces a full-scale score on the same range as the CSWT. Both tests also provide ordinal ratings (low, moderate, high). For the ordinal ratings, we use Cohen’s Kappa to assess repeatability of the ratings and validity relative to the PSS. Statistical analyses were conducted using *R Studio* with the *irr* package^26^.

### Power analysis

Sample size estimates are based on the primary analysis (test–retest reliability) using the method in^27^. We assume an expected ICC reliability of 0.75, per the definition of a clinical-grade test^18^. We set our threshold for acceptable ICC at the moderate level of 0.5. We use this lower threshold as a criterion because this is a novel test that relies on speech. Acoustic speech features inherently exhibit considerable variability, which we consider when establishing the lower performance benchmark^24^. For a significance level of 0.05 and a power of 80%, the required sample size is 55 subjects. We add an additional 5 subjects to account for potential dropouts, missing data, or issues during data collection. For the secondary analysis, a sample size of 55 subjects allows us to detect a correlation of at least 0.33 between the CSWT and PSS for a significance level of 0.05 and a power of 80%^28^.

## Data Availability

The data from this study is available and can be requested by academic researchers from the corresponding author.

## Code availability

The statistical analyses in this paper are simple (ICC, correlation, Cohen’s Kappa). A sequence of R console commands were used to generate them. This sequence of commands can be requested by academic researchers from the corresponding author.

## References

[1] Wong K, Chan AHS, Ngan SC (2019). The effect of long working hours and overtime on occupational health: A meta-analysis of evidence from 1998 to 2018. Int. J. Environ. Res. Public Health.

[2] Sara, J. D. S. *et al.* Mental Stress and Its Effects on Vascular Health. *Mayo Clin. Proc.* **97**(5), 951–990. 10.1016/j.mayocp.2022.02.004 (2022). 10.1016/j.mayocp.2022.02.004 PMC9058928 35512885

[3] Cohen S, Kamarck T, Mermelstein R (1983). A global measure of perceived stress. J. Health Soc. Behav..

[4] Roberti JW, Harrington LN, Storch EA (2006). Further psychometric support for the 10-item version of the perceived stress scale. J. Coll. Couns..

[5] Lee EH (2012). Review of the psychometric evidence of the perceived stress scale. Asian Nurs. Res..

[6] Miranda AR, Scotta AV, Méndez AL, Serra SV, Soria EA (2020). Public sector workers' mental health in Argentina: Comparative psychometrics of the perceived stress scale. J. Prevent. Med. Public Health Yebang Uihakhoe Chi.

[7] Walvekar SS, Ambekar JG, Devaranavadagi BB (2015). Study on serum cortisol and perceived stress scale in the police constables. J. Clin. Diagn. Res. JCDR.

[8] Lynch R, Flores-Torres MH, Hinojosa G, Aspelund T, Hauksdóttir A (2022). Perceived stress and hair cortisol concentration in a study of Mexican and Icelandic women. PLOS Glob. Public Health.

[9] van Marleen ME, Nicolson NA (1994). Perceived stress and salivary cortisol in daily life. Ann. Behav. Med..

[10] Ogba FN (2019). Effectiveness of music therapy with relaxation technique on stress management as measured by perceived stress scale. Medicine.

[11] Chew AMK, Ong R, Lei HH, Rajendram M, Verma SK, Gunasekeran DV (2020). Digital health solutions for mental health disorders during COVID-19. Front. Psychiatry.

[12] Slavich GM, Taylor S, Picard RW (2019). Stress measurement using speech: Recent advancements, validation issues, and ethical and privacy considerations. Stress.

[13] Voice Tool. *What is Your Level of Stress*? https://www.cignaglobal.com/stress-care/individuals/voice-tool. Accessed 8 Apr 2023 (2021).

[14] StressWaves: The World's First Voice-Activated Stress Test. *The World's First Voice-Activated Stress Test: A User's Guide*. https://www.cignaglobal.com/stress-care/employers/stress-experts/stress-waves/customers/articles/voice-activated-stress-test-user-guide. Accessed 24 Apr 2023.

[15] Hansen, J. H. & Patil, S. Speech under stress: Analysis, modeling and recognition. In *Speaker Classification I: Fundamentals, Features, and Methods*. 108–137 (2007).

[16] Cigna Global. *What is Your Level of Stress*? Cigna. https://www.cignaglobal.com/stress-care/individuals/voice-tool (2021).

[17] McCann Asia Pacific. *Cigna-StressWaves Case Study* [*Video*]. LBBOnline. https://www.lbbonline.com/work/72779. Accessed 16 Sep 2022 (2022).

[18] Fleiss JL (1999). The Design and Analysis of Clinical Experiments.

[19] Raji, I. D., Kumar, I. E., Horowitz, A. & Selbst, A. The fallacy of AI functionality. In *2022 ACM Conference on Fairness, Accountability, and Transparency*. 959–972 (2022).

[20] Shuren J, Patel B, Gottlieb S (2018). FDA regulation of mobile medical apps. JAMA.

[21] Goldsack JC, Coravos A, Bakker JP, Bent B, Dowling AV, Fitzer-Attas C, Dunn J (2020). Verification, analytical validation, and clinical validation (V3): The foundation of determining fit-for-purpose for Biometric Monitoring Technologies (BioMeTs). NPJ Digit. Med..

[22] Berisha V, Krantsevich C, Hahn PR, Hahn S, Dasarathy G, Turaga P, Liss J (2021). Digital medicine and the curse of dimensionality. NPJ Digit. Med..

[23] Berisha, V., Krantsevich, C., Stegmann, G., Hahn, S., & Liss, J. Are reported accuracies in the clinical speech machine learning literature overoptimistic? In *Proceedings of the Annual Conference of the International Speech Communication Association*, *INTERSPEECH*. Vol. 2022. 2453–2457 (2022).

[24] Stegmann GM, Hahn S, Liss J, Shefner J, Rutkove SB, Kawabata K, Berisha V (2020). Repeatability of commonly used speech and language features for clinical applications. Digit. Biomark..

[25] New Hampshire Department of Administrative Services. Perceived Stress Scale. https://www.das.nh.gov/wellness/docs/percieved%20stress%20scale.pdf. Accessed 10 Nov 2023.

[26] Gamer, M., Lemon, J., Gamer, M. M., Robinson, A., & Kendall’s, W. *Package ‘irr’. Various Coefficients of Interrater Reliability and Agreement*. Vol. 22. 1–32 (2012).

[27] Walter SD, Eliasziw M, Donner A (1998). Sample size and optimal designs for reliability studies. Stat. Med..

[28] Faul F, Erdfelder E, Lang AG, Buchner A (2007). G* Power 3: A flexible statistical power analysis program for the social, behavioral, and biomedical sciences. Behav. Res. Methods.

